# How the mapping of threshold concepts across a master’s programme in health professions education could support the development of mastersness

**DOI:** 10.1186/s12909-024-05500-4

**Published:** 2024-05-29

**Authors:** E. Archer, A. M. S. Schmutz, M. Volschenk, C. Jacobs

**Affiliations:** 1 Centre for Health Professions Education, Faculty of Medicine and Health Sciences, Stellenbosch University, Francie Van Zijl Drive, Tygerberg, 7505 Cape Town South Africa; 2 Division of Physiotherapy, Department of Health and Rehabilitation Sciences, Faculty of Medicine and Health Sciences, Stellenbosch University, Cape town, South Africa

**Keywords:** Threshold concepts, Postgraduate studies, Student support, Mastersness

## Abstract

**Introduction:**

Global trends towards the professionalization of Health Professions Education (HPE) have catalyzed the proliferation of degree-awarding programmes in HPE. We apply the theoretical lens of threshold concepts to explore the required levels of Master’s in HPE (MHPE) learning and teaching, with a view to determining how students might be supported to engage meaningfully with learning.

**Methods:**

Qualitative data were collected with a series of nominal group discussions. The methodology and data analysis followed a consensus building approach.

**Results:**

Four threshold concepts were identified: Being in the HPE world, the nature of HPE knowledge, the nature of HPE practice and the nature of HPE scholarship. We also mapped the threshold concepts to the World Federation for Medical Education (WFME) master’s level academic skills and the Scottish Quality Assurance Agency (QAA) facets of mastersness.

**Conclusion:**

It is envisaged that our findings will enhance alignment between the outcomes and assessment in an MHPE programme, form the basis of understanding feedback received from students, and inform teaching and supervisory practices. The findings also complement the WFME and QAA frameworks by clarifying the depth and complexity of academic skills expected at master’s level and informing teaching and learning approaches to support the development of the identified threshold concepts.

## Introduction

Global trends towards the professionalization of Health Professions Education (HPE) have catalyzed the proliferation of degree-awarding programmes at master’s and doctoral level in HPE [1]. During the last two decades, the master’s degree in HPE (MHPE) has emerged as the credential of choice for clinicians and biomedical scientists seeking to improve their educational effectiveness [2–4]. These programmes share the “*common goal of preparing academic leaders who are well versed in teaching, leadership, curriculum, assessment, and research through coursework, mentored experiences, and educational scholarship*” [1].

The design and implementation of MHPE programmes are well described in the literature [5, 6]. Moreover, the World Federation for Medical Education (WFME) has developed a set of Standards for Master’s Degrees in Medical and Health Professions Education, recommending that MHPE programmes should enable “*each student to develop the Master’s level academic skills of (1) independent thinking; (2) analysing, synthesising and offering a critique of information; (3) creative problem solving; (4) communicating clearly; and (5) appreciating the social, contextual and global implications of their studies and activities*” [7]. Although significant emphasis is placed on the content and outcomes of MHPE programmes [7], there are limited guidelines on the depth and complexity expected for learning at master’s level, as well as the learning and teaching approaches that might support the student in reaching the outcomes of the master’s programme. This gap is not unique to HPE and has also been identified in the wider higher education context [8–11].

Unlike many other master’s degrees, MHPE programmes do not build in linear fashion on a body of knowledge acquired during undergraduate studies, and many health professionals and biomedical scientists enrol for MHPE studies without prior educational qualifications [12]. They are further required to transition from a natural to a social sciences paradigm, thus engaging with their learning across the boundaries of two epistemologically and ontologically distinct knowledge paradigms [7]. Consequently, MHPE students may find themselves in a liminal space [13], where their self-concept, self-efficacy and worldviews are challenged [12]. The ensuing identity dissonance may influence their sense of competence and belonging as health professions educators [14]. Since research has shown that health professions educators who identify strongly with the educator role are more likely to engage in educational scholarship and leadership activities [3, 15], it is imperative that students are adequately supported in navigating the complexities of this liminal space.

The term “mastersness” is increasingly used in higher education literature to encapsulate the complexity and expectations at master’s-level study. Mastersness involves the dimensions of master’s level study, indicating the depth and level of complexity at which master’s students are required to engage with their learning. The Scottish Quality Assurance Agency (QAA) for Higher Education [16], developed a framework defining and describing seven facets of mastersness to assist lecturers and students in exploring the meaning and requirements of mastersness. These facets depict the required level of complexity; degree of abstraction; depth of learning in a subject; salience of research and enquiry; degree of learner autonomy and responsibility; complexity and unpredictability in an operational context; and professionalism that should be demonstrated at master’s level studies. However, if and how the facets of mastersness carry over to MHPE programmes is currently unclear.

The QAA facets of mastersness do not constitute learning outcomes per se, but rather “*approaches to engaging with the learning process. They are slippery concepts, open to question, but slippery concepts can be helpful if they raise student and staff awareness of what are also slippery skills levels and slippery expectations within each context” *[9]. A study by Shanley and Dalley-Hewer [8], reports that students typically refer to “light bulb” moments when they grasp what each master’s level concept entails. Due to their challenging, yet transformative nature, the facets of mastersness can arguably be conceptualised as learning thresholds. This foregrounds the notion of threshold concepts, which are considered essential for achieving proficiency in the subject and adopting the worldviews of the student’s graduate profession [17].

In this paper, we apply the theoretical lens of threshold concepts to explore the required levels of MHPE learning and teaching, with a view to determining how students might be expected and supported to engage meaningfully with learning at master’s level.

### Theoretical framing

The notion of threshold concepts (TCs) has its origin in the discipline of economics, where certain concepts were identified as key to developing proficiency in the subject [18]. Meyer and Land [13] theorised the notion of TCs as a “portal” students need to pass through or conceptual “thresholds” they need to cross within a subject to develop the required insight and understanding of the key aspects of the subject area. TCs have an innate nature of “liminality” [13, 18], which refers to the space the student occupies whilst developing proficiency in the TC. Liminality can thus be considered as an “in-between” space where learning is still taking place and proficiency not quite achieved. Meyer and Land [14] posit that TCs are transformative, irreversible, integrative, bounded, and troublesome in character. These five characteristics informed how TCs were identified in this study.

A TC is transformative in nature, as exposure to new knowledge paradigms involve both conceptual and ontological shifts. TCs are irreversible. Once a student has understood the concept, it is difficult to unlearn or forget it [13], as the concept becomes internalised. TCs are also integrative in nature and once understood, the student can connect these concepts with other concepts, thus deriving sense from previously disconnected knowledge [13].

In discipline-specific education, TCs are bounded by the discipline, for example, in our study the TCs would be bounded by knowledge specific to the field of HPE. TCs can be troublesome, as these concepts can be contested. It is therefore common for students to experience mental and emotional discomfort when they cross these conceptual “thresholds” [18].

When students experience learning challenges in a programme, it is often indicative of a TC being present. Scholars suggest that discussing and identifying TCs among subject specialists encourage conversations that could lead to better understandings of the subject area within programmes. This, in turn could assist educators to optimise curricula by structuring more time around conceptually challenging concepts [18, 19]. In addition, we addressed the important consideration of assessing the TC. As “liminality” is an “in-between” space, learning is still busy happening and therefore the assessment of each TC is complex and challenging.

We applied the theoretical lens of TCs as we sought to develop a collective understanding, from a lecturer perspective, of how students’ knowledge is shaped during MHPE studies and how they can be best supported whilst navigating the liminal spaces of learning.

The insights discussed in this section informed the methodology adopted in this study, where subject specialists across all the modules of the MHPE programme were consulted through consensus-building group discussions using a nominal group technique. Towards this end, the research question was framed as: *What are the threshold concepts underpinning the MPhil in HPE as a programme, as understood by the lecturers teaching on the programme?* Meyer and Land [13] argue that lecturers who engage with troublesome knowledge in their discipline could potentially redesign curricula to support students more effectively in making the required transitions.

### Context

**Fig. 1:**
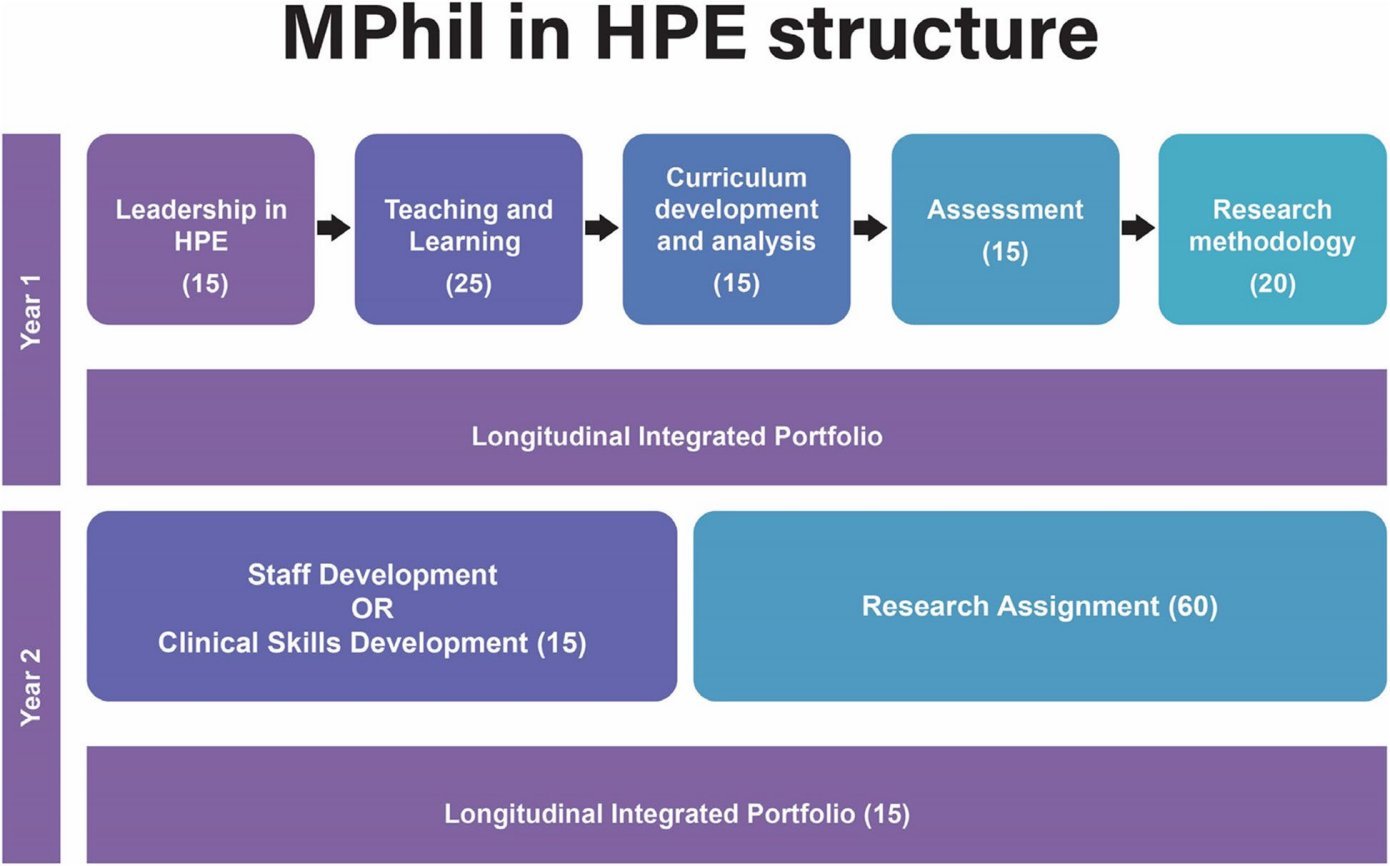
Curriculum structure of the SU MHPE programme

The Centre for Health Professions Education (CHPE) in the Faculty of Medicine and Health Sciences (FMHS) at Stellenbosch University (SU), South Africa, offers an MHPE programme that extends over a minimum of two years. The aims of the programme are to offer health professions educators the opportunity to improve their educational expertise and research skills, as well as deepen their conceptual and theoretical understanding of what it means to be a teacher at a university, specifically in the context of the health professions. Students complete all course work modules, before embarking on the Research Assignment. All modules in the programme involve two or more lecturers under the guidance of the module leader. It is thus imperative that lecturers have a congruent understanding at modular and programmatic level of the TCs that students should grasp to effectively align all curriculum activities and assessment opportunities. Figure 1 depicts the curriculum structure of the SU MHPE programme. Each block in the figure represents a different module as indicated by the module name and module credits.

Students who enrol for this programme seek to develop their academic career. They are typically employed as part- or full-time health professionals or biomedical scientists, with additional theoretical and/or clinical teaching responsibilities. Some hold joint appointments at a health service institution and the affiliated university. As mature, postgraduate students, most of these individuals have family responsibilities, which impact on the amount of time they can realistically dedicate to their studies [20].

## Methods

The study population included all 15 lecturers involved in the eight SU MHPE modules (Fig. 1), namely: Leadership in HPE; Teaching and Learning; Curriculum Development and Analysis; Assessment; Research Methodology; Staff Development (elective); Clinical Skills Development (elective); and the Longitudinal Integrated Portfolio. Those research supervisors who were external lecturers and not involved in module teaching on the MPHE programme, were excluded from the study.

**Fig. 2:**
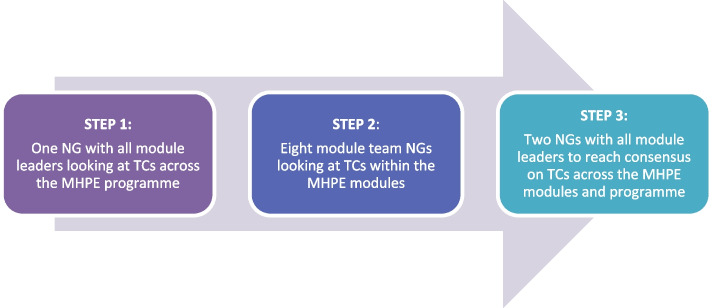
3-Step approach to data generation and analysis

The lecturers who participated in the study were viewed as contributors and co-creators of knowledge. Drawing on the notion of “best collective judgement” [21], we followed a consensus building approach to data gathering, recognising that the data may be open to consideration for change as other perspectives may oppose it. Data was generated using the nominal group (NG) technique [22] across multiple groups (*n* = 11) in a three-step process (Fig. 2). This iterative process involved discussions towards reaching collective understanding and reasonable agreement on TCs pertaining to the MHPE programme at SU [21].

The Nominal Group Technique (NGT) is a structured method performed in a group setting whereby people generate suggestions, prioritize them, and then make decisions [22, 23]. The participants who were part of the process were all lecturers involved in the MHPE programme, who had the relevant expertise to contribute meaningful to the conversations. The NGT’s method allowed the facilitator to follow the various steps starting with silent idea generation, then a round robin sharing of everyone’s ideas, followed by a discussion and then requesting the lecturers to rank the listed ideas according to perceived importance.

As a first step, a NG was conducted with the module leaders to identify the overarching programme TCs, the second step involved a NG conducted with each of the eight module teams to identify the TCs at modular level, and then the third step, two NGs were conducted with only the module leaders with the aims of reaching consensus and prioritizing the module level TCs identified during step 2.

Participants were invited to the NGs via email. Participation was voluntary and informed consent was obtained prior to data generation. NGs were scheduled according to the availability of participants over an extended period of about two years due to the disruptions caused by the COVID-19 pandemic. NGs were initially conducted in person but were moved online via Microsoft Teams during lockdown restrictions. The nominal groups were facilitated by one of the researchers.

### Process followed for each of the nominal groups

A four-phased approach was followed for each of the eleven NGs with a view to reaching a shared understanding of the notion of TCs; collectively brainstorming and clustering TCs; reaching consensus on TCs across the entire MHPE programme; and prioritizing the identified TCs.

At the start of each NG, the facilitator introduced the aim of the study and defined the term TCs. During the first stage, participants were requested to engage in a ‘silent generation of ideas’ for about ten minutes [23], where they would think about possible TCs pertaining to the programme and/ or the module that they were involved in.

In the second stage, each participant was invited to share their ideas with the rest of the group. All ideas mentioned were listed by the facilitator. Stage three involved a brief discussion of each of these ideas to facilitate clarification and prevent duplication.

During the fourth and last stage participants were requested to vote and rank the identified TCs.

The four-phased process was iterative as each step in the data gathering process fed into the next. The importance of the process of “meaning making” was facilitated at each step through collective brainstorming and clustering, consensus-reaching and prioritizing, followed by a mapping of the TCs so that deep contextual understandings of these TCs were developed. At each stage ideas were discussed at length until a point of collective consensus was achieved.

## Results

The mapping of participants’ ideas into four TC clusters was achieved collectively and when ideas differed, they were debated until all participants were satisfied with the inclusion in the TC cluster. Only ideas on which collective consensus was reached were included. The descriptions of each of the four TC clusters were discussed. Differences of opinion were debated at length until a point of collective consensus was achieved through voting. Those views on which consensus could not be reached were omitted by mutual agreement.

Collective consensus was reached on four TCs for the SU MHPE programme: (1) Being in the HPE world, (2) The nature of HPE knowledge, (3) The nature of HPE practice, and (4) The nature of HPE scholarship. These four TCs were refined and prioritized across steps 1, 2 and 3 of the data generation process, resulting in the following collective descriptions (Supplemental Appendix A).

1. Being in the HPE world
  This TC describes how “being in the HPE world” is explicitly expressed through students’ worldviews (epistemology and ontology) and how they can use theory not simply as a concept but also as a model of explanation to articulate their worldview.
2. The nature of HPE knowledge
  This TC represents students’ understanding of the complexity of HPE knowledge, and how they link such understandings to conceptions of learning.
3. The nature of HPE practice
  This TC involves students’ understanding of HPE practice and the various philosophies and conceptions of teaching.
4. The nature of HPE scholarship
  Students explicitly explore HPE scholarship concepts to evaluate their current epistemological stance, by taking an informed position and reflecting on choices they make during the development of their research protocol.

**Fig. 3:**
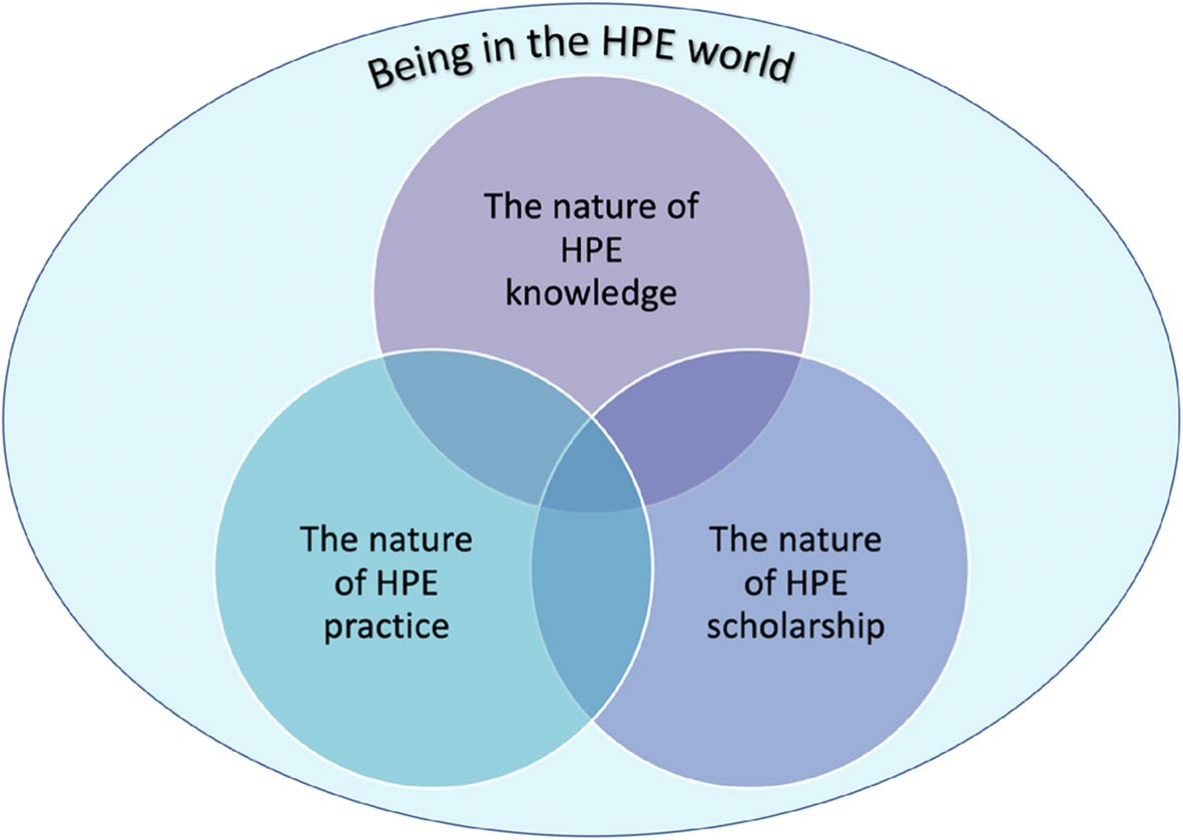
Relationality of the four SU MHPE TCs

Participants furthermore agreed upon the relationality of these four TCs. TC1 was regarded as a meta-TC,^1^ as it was of a philosophical nature. TC1 was thus regarded as an overarching TC, while TCs 2, 3 and 4 were deemed relational, as depicted by the intersecting Venn circles in Fig. 3 below.

The mapping of these four TCs across the MPHE programme revealed that two modules, namely Leadership in year 1, and Staff Development in year 2, did not overtly include TC4 (The nature of HPE scholarship) as a learning activity. While this may point to a potential gap in the development of TC4 at the start of the first and second years of the programme, it could also be argued that this omission may limit cognitive overload. The Integrated Portfolio module was the only module that appeared to cover all four TCs overtly as learning activities. Some examples of learning activities for the TCs in various modules are offered below (Table 1).

**Table 1:** Examples of learning activities for the TCs in various MHPE modules

TC	MODULE	LEARNING ACTIVITY
**TC1** **Being in the HPE world**	Leadership	Myers Briggs Type Indicator exercise helps to create awareness of own and others’ personality styles as leaders
**TC2** **The nature of HPE knowledge**	Teaching and Learning	Students discuss the relationship/ interconnectedness between teaching and learning and outcomes and assessment
**TC3** **The nature of HPE practice**	Curriculum	Students are introduced to different conceptions of curriculum models (didactic lecture) which they are required to apply to their own curriculum (classroom exercise)
**TC4** **The nature of HPE scholarship**	Research Methodology	Students explicitly explore HPE scholarship concepts to evaluate their current epistemological stance Checkpoints are designed throughout the module where students take an informed position and are encouraged to reflect on choices they make during the development of research protocol

Mapping of assessment opportunities across the programme revealed that, although most modules assessed TC1, TC3 and TC4, only four modules provided an assessment opportunity for TC2 (The nature of HPE knowledge). The challenges related to assessing TC2 suggest a potential gap in the assessment strategies across the programme. Some examples of assessment opportunities for the TCs in various modules are offered below (Table 2).

**Table 2:** Examples of assessment opportunities for the TCs in various MHPE modules

TC	MODULE	ASSESSMENT OPPORTUNITY
**TC1** **Being in the HPE world**	Leadership	Situational analysis to critique specific leadership style in own educational environment and select leadership style to manage contextualised educational change issue
**TC 2** **The nature of HPE knowledge**	Integrated Portfolio	e-Portfolio pages must include evidence of regular critical reflection, insights into the impact of their own learning on their evolving teaching philosophy and teaching practice, development areas identified and professional development planning and evaluation. A final reflective narrative pulls all threads together
**TC3** **The nature of HPE practice**	Curriculum	The assessment task requires students to critically reflect on their own curriculum and this reflection is assessed
**TC4** **The nature of HPE scholarship**	Research Methodology	Research projects focus on HPE so students’ understanding of key HPE concepts is captured and assessed within their protocols

Knowledge areas in which students typically struggled were also explored during the final NG. While TCs 1, 2 and 3 were identified as areas of difficulty by several modules, TC 4 (The nature of HPE scholarship) was identified as the concept that students found most challenging in seven of the eight modules. This could be attributed to the fact that most MHPE students need to transition from a natural science to a social sciences paradigm [7]. This may require significant epistemological and ontological shifts for most students. Although all the TCs were identified and mapped across the teaching and learning activities and assessment opportunities of the MHPE programme, the Integrated Portfolio was the only module that directly addressed all four TCs. It could, therefore, be argued that longitudinal modules, such as the Integrated Portfolio*,* might best enable students to navigate the liminal space towards mastering the identified TCs.

## Discussion

Our findings support research on master’s level studies in the wider higher education context, which shows that taught master’s programmes constitute a complex and multifaceted learning journey, and that, like students, lecturers may find it challenging to conceptualise the meaning and requirements of “mastersness” [9]. This, in turn, may influence the ways in which MHPE students are supported in the learning process.

The notion of TCs, as well as the guidelines provided by the QAA “mastersness” framework and the WFME standards enabled the SU MHPE lecturers to engage in more complex ways with what is expected of our students. The four TCs identified in this study illuminated the researchers’ and lecturers’ conceptions of the required levels of learning and teaching at master’s level in the MPhil in HPE programme. The overarching TC1 *(Being in the HPE world)* reflects the tensions that most MHPE students experience when transitioning from a natural science to a social science ontology. The resulting dissonance often poses a significant barrier to their learning, progression and understanding of the field of HPE. The interlinking TCs 2, 3 and 4 (*The nature of HPE knowledge, practice, and scholarship*) illuminate the challenges that MHPE students experience in relation to the unfamiliar scholarship conventions of the new knowledge paradigm where they are required to navigate their professional learning, practice, and identity.

**Fig. 4:**
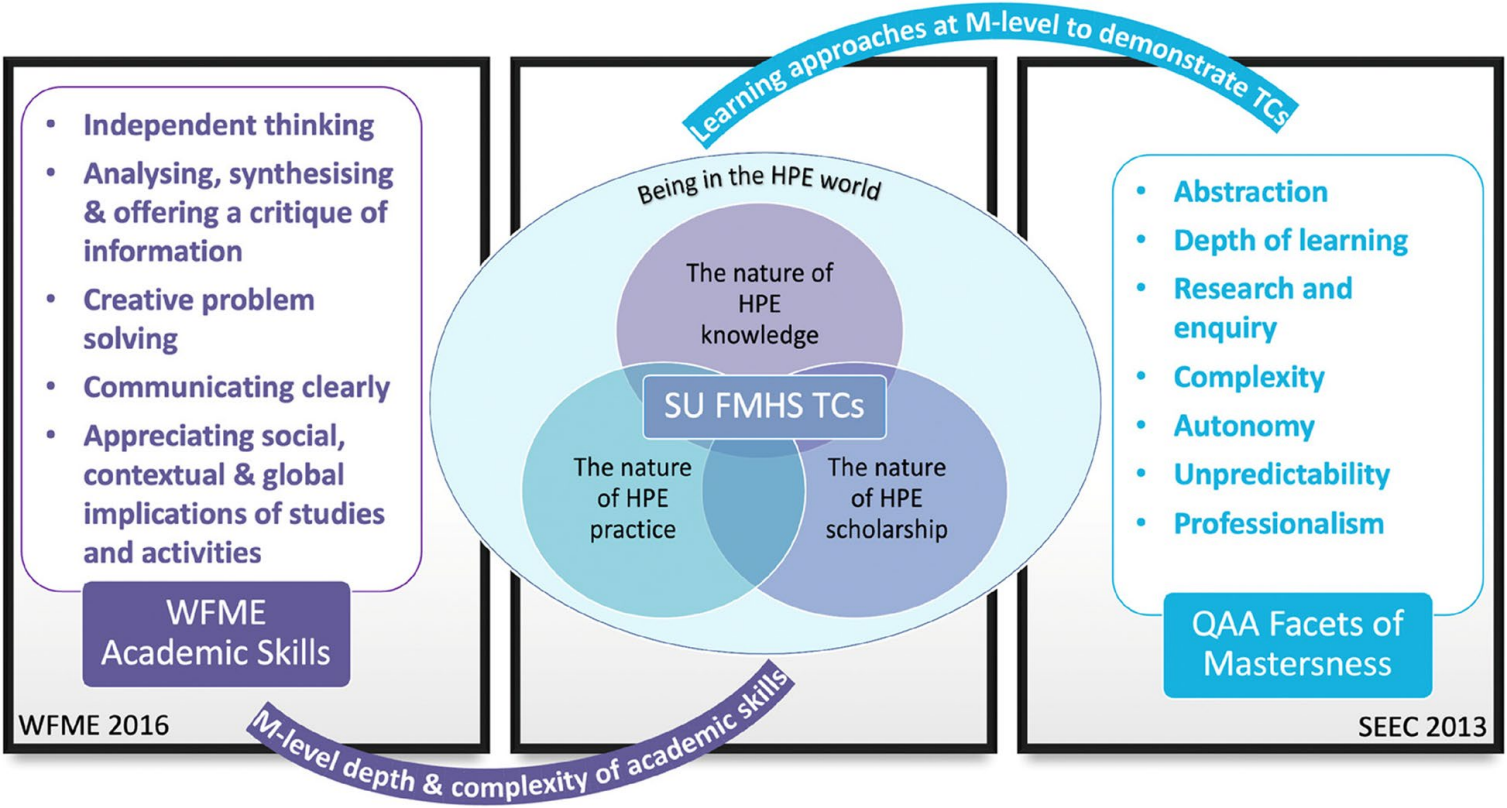
Mapping of MHPE TCs to WFME Academic Skills [7], and QAA Facets of Mastersness [16]

As discussed in the introduction, the WFME stipulates five academic skills that MHPE students need to develop, but the depth and complexity of academic skills expected at master’s level are not specified [7]. Moreover, it was not evident if and how the seven QAA facets of mastersness [16], which depict approaches to engaging with the learning process at master’s level, were transferable to MHPE studies. It was further not clear how these two frameworks could be used to support learning and teaching in MHPE programmes. Since TCs serve to illuminate the key constructs that underpin the mastery of a specific subject area, the researchers mapped the four SU MHPE TCs in relation to the five WFME master’s level academic skills [7], and the seven QAA facets of mastersness to explore possible relationships (Supplemental Appendix B) [16]. We found that the SU MHPE TCs complemented guidelines and frameworks that were developed at a more global level. Not only did the four TCs prove helpful in clarifying the depth and complexity at which academic skills should ideally be developed in MHPE programmes [7], but the seven facets of mastersness foregrounded important approaches to teaching and learning that may potentially support MHPE students during master’s level study (See Fig. 4) [17].

Further research should explore if and to what extent the QAA facets of mastersness [16], need to be adapted for HPE to ensure that approaches to teaching and learning in MHPE programmes effectively address the unique complexities of navigating learning within the liminal space between two epistemologically and ontologically distinct knowledge paradigms.

Practical implications of the above include the recognition that MHPE studies constitute a transformative, yet challenging learning experience. Globally, there are limited guidelines and frameworks to address the depth and complexity of master’s level learning, specifically in HPE. The mapping of TCs in an MHPE programme provides new insights into the expectations of, and support for, student learning at master’s level. The mapping from our study fills a gap that complements and extends the MFME and QAA guidelines.

### Limitations and future considerations

We acknowledge that TCs are contextual and in this study, they were co-created by all the lecturers involved in the MHPE at SU. The voice of the students was not included in this study and should be explored in future research to complement the findings in this study.

## Conclusion

The framework (Fig. 3) arising from the findings offers a point of departure for other MHPE programmes engaging with what it means to be and become a Master in HPE. The results of this study could form the basis of understanding the challenges MHPE students face with TCs, which could assist lecturers to improve and strengthen teaching within MHPE programmes, with a view to supporting students in their learning process. The results of the mapping process (Fig. 4) also yielded insights on mastersness, which complements the rather limited guidelines and frameworks provided globally. The four TCs emerging from this study, extend the meta level WFME Academic Skills guidelines [7], and the QAA Facets of Mastersness framework [16], by clarifying the depth and complexity of the WFME academic skills, and by demonstrating how the seven facets of mastersness could inform teaching and learning approaches to support the TCs. This mapping offers a detailed framework to guide MHPE programme committees in ensuring that TCs are appropriately scaffolded through learning activities and assessment opportunities at all levels of MHPE programmes. The mapping process also raises issues about the curriculum structure of MHPE programmes. Based on the results of our study, we would recommend the inclusion of a longitudinal module (such as the *Integrated Portfolio*) which could form the vertical spine of MHPE programmes and which could become the curriculum space through which the TCs are developmentally scaffolded. Such a module could also provide the “golden thread” for the TCs, as well as a longitudinal structure linking all the other modules and providing curriculum coherence.

## Supplemental Appendix A: MPhil TCs with descriptions

**Table:** 

1. **BEING IN THE HPE WORLD**	2. **THE NATURE OF HPE KNOWLEDGE**	3. **THE NATURE OF HPE PRACTICE**	4. **THE NATURE OF HPE SCHOLARSHIP**
**Worldview** • *Explicitness of worldview* • *Reflexivity (awareness of self)* **Epistemology & Ontology** • *Social responsiveness (philosophy)* • *Paradigms (shift from biomedical model)* • *Knowledge production (skill)* **Theory** • *Theory (as a notion/concept) – E.g.: self-directed/regulated learning; transformative learning; CoPs …* • *Theory (as a model of explanation)—reasoning using theory*	**HPE** (as a body of knowledge) • *What it looks like* • *Complexity* • *Not one way (not absolute, not binary)* • *Uncertainty* • *Alternative ways of thinking (envisioning)* • *Interconnectedness* **Conceptions of learning** • *education as a concept*	**Ways of thinking about T&L** (Conceptions of teaching) • *Teaching vs development* • *Facilitation of learning* • *Reflective practice* • *Feedback (reflection)* • *Assessment of, for and as learning* • *Constructive alignment* **Philosophy of teaching**	**Criticality** (developing it) • *Critical (academic) reflection* • *Critique (evaluation)* • *Critical disposition* • *Questioning* **Argumentation** • *Argument (rationale)* • *Synthesis* • *Academic writing (putting together)* • *Recognising the line of enquiry* **Taking an informed position** • *Research design (alignment)* • *Meaning of data (interpretation)* • *Making inferences* • *Abstraction*
TC 1 is a meta-TC and is of a philosophical nature. It overarches the other three TCs, as indicated in the diagram The other three TCs (2, 3 and 4) are relational, as represented by the intersecting Venn circles in the diagram			
This TC is about how *‘being in the HPE world’* is explicitly expressed through students’ worldview (epistemology and ontology) and how they are able to use theory not simply as a concept but also as a model of explanation to articulate their worldview	This TC is about how students understand the complexity of HPE knowledge, and how they link these understandings to conceptions of learning	This TC is about how students understand HPE practice and the various philosophies and conceptions of teaching	This TC is about how students understand HPE scholarship, particularly what it means to take an informed position supported by argumentation and criticality

## Supplemental Appendix B: Mapping SU MHPE TCs to WFME Academic Skills (WFME 2016) and QAA Facets of Mastersness (SHEEC 2013)

**Table:** 

**WFME: Master’s level academic skills** Intellectual, personal, and professional abilities (WFME 2016, p. 9)	**SU MHPE Threshold concepts** Conceptual ‘thresholds’ students need to cross within a subject to develop the required insight and understanding to proceed through and master aspects of the subject area. Includes ontological shifts	**QAA: Facets of Mastersness** Approaches to engaging with the learning process (SHEEC 2013, p. 3–9)
**WFME 1:** Independent thinking **WFME 5:** Appreciating the social, contextual, and global implications of their studies and activities	**SU MHPE TC 1:** Being in the world *(How ‘being in the HPE world’ is explicitly expressed through students’ worldview (epistemology and ontology) and how they are able to use theory not simply as a concept but also as a model of explanation to articulate their worldview)* **Worldview** • *Explicitness of worldview* • *Reflexivity (awareness of self)* **Epistemology & Ontology** • *Social responsiveness (philosophy)* • *Paradigms (shift from biomedical model)* • *Knowledge production (skill)* **Theory** • *Theory (as a notion/concept) – E.g.: self-directed/regulated learning; transformative learning; CoPs …* • *Theory (as a model of explanation)—reasoning using theory*	**Mastersness facet 1:** Abstraction *Extracting knowledge or meanings from sources and then using these to construct new knowledge or meanings* **Mastersness facet 5:** Autonomy *Taking responsibility for own learning* • *self-organization* • *motivation* • *location and acquisition of knowledge* **Mastersness facet 7:** Professionalism *Displaying appropriate professional attitudes, behavior and values* • *learning ethical behaviors* • *developing academic integrity* • *dealing with challenges to professionalism* • *recognizing the need to reflect on practice* • *becoming part of a discipline/occupational community*
**WFME: Master’s level academic skills** Intellectual, personal, and professional abilities (WFME 2016, p. 9)	**SU MHPE Threshold concepts** Conceptual ‘thresholds’ students need to cross within a subject to develop the required insight and understanding to proceed through and master aspects of the subject area. Includes ontological shifts	**QAA: Facets of Mastersness** Approaches to engaging with the learning process (SHEEC 2013, p. 3–9)
**WFME 1**: Independent thinking **WFME 3**: Creative problem solving **WFME 5**: Appreciating the social, contextual and global implications of their studies and activities	**SU MHPE TC 2:** The nature of HPE knowledge *(How students understand the complexity of HPE knowledge, and how they link these understandings to conceptions of learning)* **HPE** (as a body of knowledge) • *What it looks like* • *Complexity* • *Not one way (not absolute, not binary)* • *Uncertainty* • *Alternative ways of thinking (envisioning)* • *Interconnectedness* **Conceptions of learning** • *education as a concept*	**Mastersness facet 1:** Abstraction *Extracting knowledge or meanings from sources and then using these to construct new knowledge or meanings* **Mastersness facet 2:** Depth of learning *Depth of learning* • *acquiring more knowledge* • *using knowledge differently* **Mastersness facet 4:** Complexity *Recognizing and dealing with complexity of knowledge* • *integration of knowledge and skills* • *application of knowledge in practice* • *conceptual complexity* • *complexity of learning process* **Mastersness facet 5:** Autonomy *Taking responsibility for own learning* • *self-organization* • *motivation* • *location and acquisition of knowledge* **Mastersness facet 6:** Unpredictability *Dealing with unpredictability in operational contexts* • *recognizing that 'real world' problems are by their nature 'messy' and complex* • *being creative with the use of knowledge and experience to solve these problems*
**WFME: Master’s level academic skills** Intellectual, personal, and professional abilities (WFME 2016, p. 9)	**SU MHPE Threshold concepts** Conceptual ‘thresholds’ students need to cross within a subject to develop the required insight and understanding to proceed through and master aspects of the subject area. Includes ontological shifts	**QAA: Facets of Mastersness** Approaches to engaging with the learning process (SHEEC 2013, p. 3–9)
**WFME 1**: Independent thinking **WFME 5**: Appreciating the social, contextual and global implications of their studies and activities	**SU MHPE TC 3:** The nature of HPE practice *(How students understand HPE practice and the various philosophies and conceptions of teaching)* **Ways of thinking about T&L** (Conceptions of teaching) • *Teaching vs development* • *Facilitation of learning* • *Reflective practice* • *Feedback (reflection)* • *Assessment of, for and as learning* • *Constructive alignment* **Philosophy of teaching**	**Mastersness facet 1**: Abstraction *Extracting knowledge or meanings from sources and then using these to construct new knowledge or meanings* **Mastersness facet 4**: Complexity *Recognizing and dealing with complexity of knowledge* • *integration of knowledge and skills* • *application of knowledge in practice* • *conceptual complexity* • *complexity of learning process* **Mastersness facet 5**: Autonomy *Taking responsibility for own learning* • *self-organization* • *motivation* • *location and acquisition of knowledge* **Mastersness facet 7**: Professionalism *Displaying appropriate professional attitudes, behavior and values* • *learning ethical behaviors* • *developing academic integrity* • *dealing with challenges to professionalism* • *recognizing the need to reflect on practice* • *becoming part of a discipline/occupational community*
**WFME: Master’s level academic skills** Intellectual, personal, and professional abilities (WFME 2016, p. 9)	**SU MHPE Threshold concepts** Conceptual ‘thresholds’ students need to cross within a subject to develop the required insight and understanding to proceed through and master aspects of the subject area. Includes ontological shifts	**QAA: Facets of Mastersness** Approaches to engaging with the learning process (SHEEC 2013, p. 3–9)
**WFME 1**: Independent thinking **WFME 2**: Analyzing, synthesizing and offering a critique of information **WFME 3**: Creative problem solving **WFME 4**: Communicating clearly	**SU MHPE TC 4:** The nature of HPE scholarship *(How students understand HPE scholarship, particularly what it means to take an informed position supported by argumentation and criticality)* **Criticality** (developing it) • *Critical (academic) reflection* • *Critique (evaluation)* • *Critical disposition* • *Questioning* **Argumentation** • *Argument (rationale)* • *Synthesis* • *Academic writing (putting together)* • *Recognising the line of enquiry* **Taking an informed position** • *Research design (alignment)* • *Meaning of data (interpretation)* • *Making inferences* • *Abstraction*	**Mastersness facet 1**: Abstraction *Extracting knowledge or meanings from sources and then using these to construct new knowledge or meanings* **Mastersness facet 3**: Research and enquiry *Developing critical research and enquiry skills & attributes* **Mastersness facet 4**: Complexity *Recognizing and dealing with complexity of knowledge* • *integration of knowledge and skills* • *application of knowledge in practice* • *conceptual complexity* • *complexity of learning process* **Mastersness facet 5**: Autonomy *Taking responsibility for own learning* • *self-organization* • *motivation* • *location and acquisition of knowledge*

## Data Availability

The data can be made available if requested via the corresponding author.

## Footnotes

1 The term meta-TC was coined by the authors to indicate the philosophical nature of this TC.
